# Removal of Hepatitis B virus surface HBsAg and core HBcAg antigens using microbial fuel cells producing electricity from human urine

**DOI:** 10.1038/s41598-019-48128-x

**Published:** 2019-08-13

**Authors:** Grzegorz Pasternak, John Greenman, Ioannis Ieropoulos

**Affiliations:** 1 Bristol BioEnergy Centre, Bristol Robotics Laboratory, University of the West of England, Coldharbour Lane, BS16 1QY Bristol, UK; 2 Laboratory of Microbial Electrochemical Systems, Department of Polymer and Carbon Materials, Faculty of Chemistry, Wroclaw University of Science and Technology, Wyb. Wyspiańskiego 27, 50-370 Wrocław, Poland

**Keywords:** Environmental biotechnology, Environmental impact, Chemical engineering, Bioenergy

## Abstract

Microbial electrochemical technology is emerging as an alternative way of treating waste and converting this directly to electricity. Intensive research on these systems is ongoing but it currently lacks the evaluation of possible environmental transmission of enteric viruses originating from the waste stream. In this study, for the first time we investigated this aspect by assessing the removal efficiency of hepatitis B core and surface antigens in cascades of continuous flow microbial fuel cells. The log-reduction (LR) of surface antigen (HBsAg) reached a maximum value of 1.86 ± 0.20 (98.6% reduction), which was similar to the open circuit control and degraded regardless of the recorded current. Core antigen (HBcAg) was much more resistant to treatment and the maximal LR was equal to 0.229 ± 0.028 (41.0% reduction). The highest LR rate observed for HBsAg was 4.66 ± 0.19 h^−1^ and for HBcAg 0.10 ± 0.01 h^−1^. Regression analysis revealed correlation between hydraulic retention time, power and redox potential on inactivation efficiency, also indicating electroactive behaviour of biofilm in open circuit control through the snorkel-effect. The results indicate that microbial electrochemical technologies may be successfully applied to reduce the risk of environmental transmission of hepatitis B virus but also open up the possibility of testing other viruses for wider implementation.

## Introduction

Microbial electrochemical technology encompasses several types of bioelectrochemical systems, including but not limited to microbial fuel cell (MFC) and microbial electrochemical snorkels. It is attracting increasing attention in recent years both from the research and industrial communities^1^. MFC and electrochemical snorkel are technologies where the bioelectrochemical reactor converts organic matter into electricity with the use of electroactive bacteria. In its most conventional setup, the MFC consists of an anode, a cathode and a separator dividing the two half-cells^2^. Significant effort has been made in the last few years to improve the energy efficiency of MFCs in terms of the structural elements: anode^3,4^, cathode^5,6^, separator^7–11^, as well as overall design improvements and operational conditions^12–15^. In contrast, the snorkel-reactors are bridging two redox zones with one electrode serving both as the anode and cathode^16,17^.

Two of the most promising areas of practical implementation for MFCs are remote power for low-power applications as well as wastewater treatment, while microbial electrochemical snorkel technology is most promising in bioremediation purposes^18^. Several pilot-scale studies have been successfully carried out to convert various types of waste such as urine, domestic and food industry wastewater, into useful energy^19–21^. Such an approach may supplement the other emerging techniques for sustainable use of resources^22,23^. Nevertheless, development of new-generation wastewater treatment techniques requires an extensive range of studies on health hazards related to the potential release of infectious disease vectors into the MFC treatment systems, which could propagate out to the environment. So far this issue has been less studied^24,25^.

Recent work in this area specifically, has shown new findings on the fate of pathogenic bacteria in MFC systems. These results led to the first conclusions on the feasibility of biological stabilisation of the MFC effluents. In these previous studies it has been shown that over 99.99% of pathogenic *Salmonella enteritidis* were removed from neat human urine flowing through a MFC cascade system. Moreover, none of the pathogenic bacteria were adsorbed by the electroactive biofilm^26^. Similar results were achieved for the other pathogenic species^27^. Previous research indicated, that hydrogen peroxide, a byproduct of oxygen reduction reaction at the cathode may be used to significantly reduce the number of total coliforms in a wetland system^28^. A combined approach to increase the cathodic efficiency and disinfect anolyte through the recirculation of catholyte was carried out by using sodium hypochlorite by Jadhav *et al*.^28^. An *in-situ* MFC stack has also recently been reported to reduce the numbers of faecal and total coliforms coming from neat human waste by Cid *et al*.^29^.

Although some progress has been made to evaluate the sanitary risk related to the pathogenic bacteria present in MFC systems, none of the studies in the field have addressed the survival of enteric viruses in such bioelectrochemical reactors and yet their presence in the environment is directly related to faecal pollution. More than 100 types of virus may be discharged to freshwater reserves with animal and human waste^30^. Moreover, it has been shown, that urine may be regarded as a potential route of transmission of hepatitis B virus (HBV, Hep B)^31^. According to recent estimates, more than 240 million people worldwide are infected with HBV which may lead to liver failure as well as liver cancer^32,33^.

**Figure 1:**
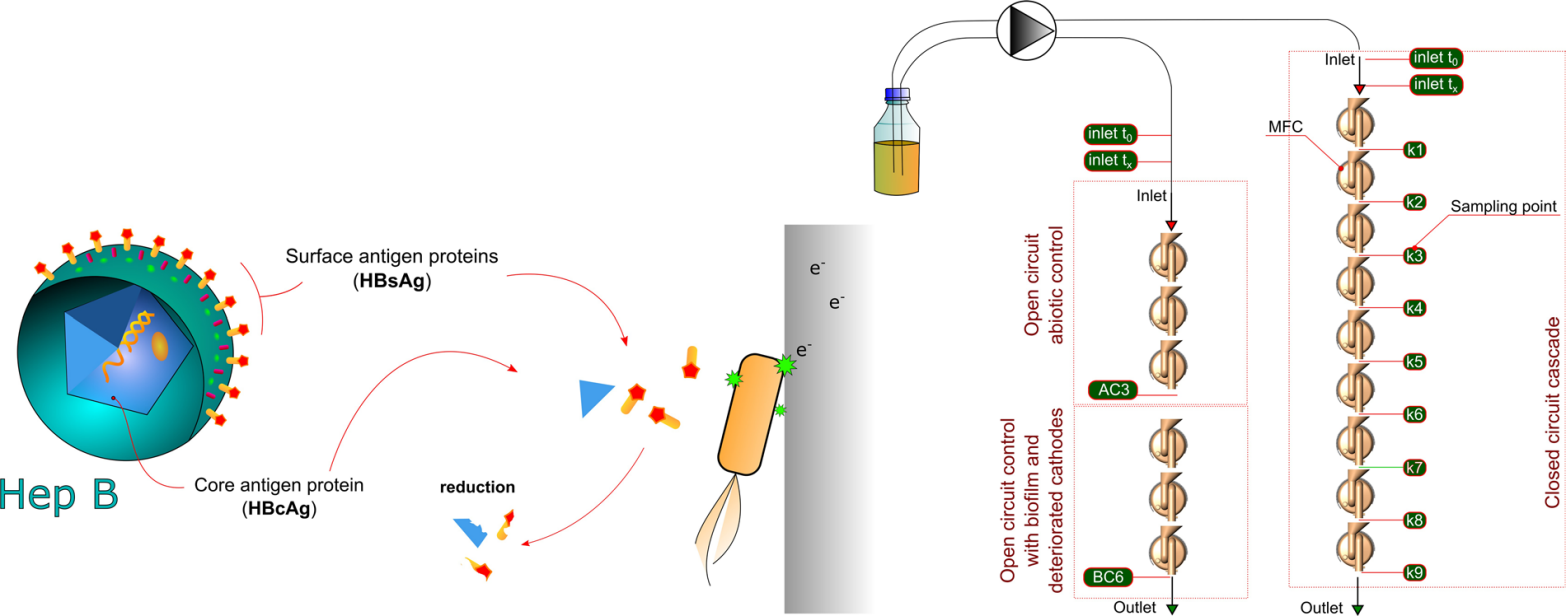
Schematic representation of the experimental setup and structure of Hepatitis B virus with its elements (HBcAg and HBsAg) that were deactivated in MFCs. Graphical elements of the MFC setup were derived from our previous works^26,40^.

It is known that disease vectors may be transmitted from the wastewater systems either through discharge or emission of aerosols^34^. Therefore, it is crucial to study the survival rate of enteric viruses in order to fully evaluate the sanitary risks and required operational conditions in all novel sanitation technologies. In this study, we have chosen the Hep B virus as a model organism for this purpose, and used two of its crucial structural components for the investigation: surface antigen (HBsAg) and core antigen (HBcAg) proteins. The surface antigen forms the envelope, which is surrounding the capsid, composed of the core antigen (Fig. 1). Damaging these components may prevent the adsorption to the host and inactivation of virus replication^35^. Both of these antigens were thus studied as surrogates for determining the susceptibility of HBV and enteric viruses to different inactivation factors such as heat, disinfectants or proteolytic enzymes^36–38^.

Our aim in the current study was therefore to investigate for the first time, the survival of pathogenic hepatitis B virus in microbial fuel cell systems and to outline the principal hydraulic and electrochemical conditions required to reach the required disinfection and inactivation efficacy of the virus.

## Results and Discussion

**Figure 2:**
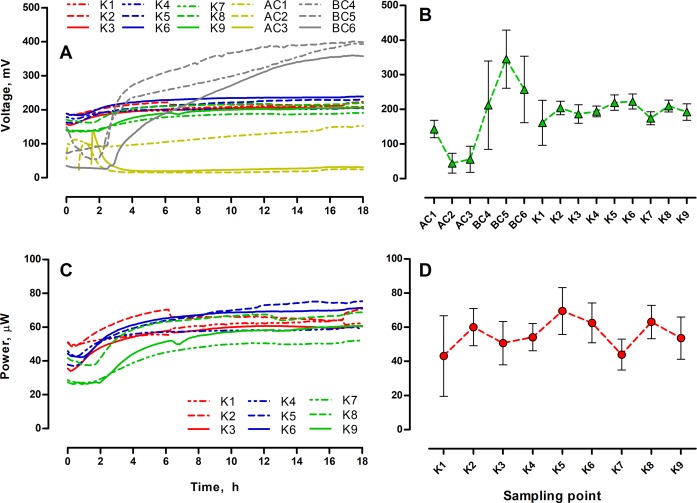
Real time potential (**A**), power output (**C**) and the corresponding average values calculated for the whole experimental period ± SD (**B**,**D**). Data represent average ± SD.

The experimental setup consisted of 9 MFCs operating in closed circuit (CC) mode and 6 MFCs operating in open circuit (OC) mode as two individually-fed cascades (Fig. 1). Real time power performance was monitored during the whole experimental period (Fig. 2). The CC-MFCs displayed stable potential between 161–222 mV. The lowest potential was recorded for OC-AC (abiotic control) MFCs and reached a minimum of 44.4 ± 28.8 mV. The OC-BC (biotic control) MFCs displayed much higher OCV values when compared to OC-AC values. The OCV recorded for biotic control was within the range of 211–345 mV. Such a difference indicated that the biofilm developed at the anodic surface of BC-MFCs, was metabolically active and affected the overall OC-MFCs potential. The potential is known to be a major factor affecting the biofilm function and activity^39^. Furthermore, it is very likely that during operation, the OC-MFCs have acted as a microbial electrochemical snorkel leading to increased electroactivity of the biofilm, although no current was recorded (via the resistor circuit) in this mode.

Power output observed in CC-MFCs ranged between minimum and maximum values of 43.2 and 69.5 µW for the 1^st^ and 5^th^ MFCs in the cascade, respectively. Such values are lower than those previously observed for the same type of MFCs due to underperformance of the cathode electrodes through long term use and slow deterioration^15,40^.

**Figure 3:**
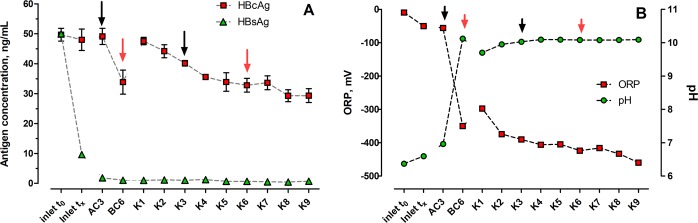
(**A**) Changes in core (HBcAg) and surface (HBsAg) antigen concentrations when treated in the MFC cascade. (**B**) Changes in redox potential and pH along MFC cascade setup. Red and black arrows indicate two corresponding MFCs in control open circuit cascade and closed circuit cascade. For clarity, only fluidically neighbouring points of the MFC setup have been connected by dashed lines. Data represent average ± SD.

The trials for inactivation of hepatitis B surface (HBsAg) and core (HBcAg) antigens revealed their different susceptibility for degradation in MFC setups (Fig. 3A). The surface antigen was almost entirely inactivated once it was introduced to either CC and OC MFCs. Its concentration had already decreased from 50 to 9.64 ± 0.45 ng/mL prior to entering the MFC cascades. Such results suggest that only a slight decrease in pH and ORP potential (Fig. 3B), along with time as another factor, were sufficient for the efficient inactivation of surface antigen due to its instability. Previous research has indicated that HBsAg is susceptible for inactivation by using various physical and chemical factors such as heat, ethanol, glutaraldehyde and several plant extracts^36,41^ as well as enzymatic degradation^42^.

In contrast, treatment of the core antigen in the MFC cascades resulted in stepwise, nearly linear decrease of its concentration in subsequent CC-MFCs. The HBcAg concentration decrease started from 47.59 ± 1.25 ng/mL, observed for K1-MFC and reached 29.33 ± 2.32 ng/mL, observed for K9. Although the core antigen was also susceptible to inactivation within the MFC environment, it was much more resistant than surface antigen.

Monitoring of pH and ORP indicated significant differences between AC and BC controls. Only a slight increase of urine pH was observed for the abiotic control (from 6.60 to 6.96), while significant increase of pH (to 10.12) was noted for the biotic control BC-MFCs. A similar trend was observed for ORP where the potential changed from −50.0 to −55.7 mV for AC and to −350.0 mV for BC MFCs. Therefore, the ORP observed for the biotic control was within the range of ORP observed for CC MFCs (−297.7 mV to −460.0 mV). Such low ORP values in BC MFCs suggested the occurrence of either methanogenic or electroactive reactions^43,44^, on the assumption of possible short-circuiting of the two electrodes through the porous earthenware separator for the latter case.

Calculated surface antigen LR values revealed only negligible differences in absolute numbers between AC, BC and CC MFCs (Table 1). Some increase of LR for CC MFCs was noticed when comparing AC3 (1.43 ± 0.11) and K3 (1.68 ± 0.10) as well as BC6 (1.69 ± 0.09) to K6 (1.86 ± 0.20). Nevertheless, none of these differences was statistically significant, as confirmed by the t-test analysis.

**Table 1:** Log reduction (LR) of surface and core antigens in MFC cascade system and the results of statistical significance of the difference between LR average values.

Type of antigen	Parameter	Control MFCs (OC)		Closed circuit MFCs (CC)		
		Abiotic (AC3)	Biotic (BC6)	K3	K6	K9
	Position in cascade	3	6	3	6	9
HBsAg	LR ± SD	1.43 ± 0.11	1.69 ± 0.09	1.68 ± 0.10	1.86 ± 0.20	1.84 ± 0.05
	t-test p-value		0.16	0.18	0.38	
	t-test conclusion		BC6 = AC3	K3 = AC3	K6 = BC6	
HBcAg	LR ± SD	0.005 ± 0.023	0.167 ± 0.041	0.093 ± 0.017	0.180 ± 0.026	0.229 ± 0.028
	p-value		0.03^a^	0.02^a^	0.70^b^	
	test conclusion		BC6 > AC3	**K3** **>** **AC3**	K6 = BC6	

^*^LR – Log Reduction, SD – Standard deviation, ^a^results of t-test, ^b^results of Wilcoxon-Matt-Whitney test.

The LR values observed for core antigen were approximately one log-fold lower, reaching a maximum value of 0.229 ± 0.028 observed at the end of CC cascade (K9). Furthermore, a significant statistical difference was observed between the AC3 and K3 MFCs, as well as between AC3 and BC6 open circuit controls. Although the LR value observed for BC6 was lower than that of CC MFC (K6), no significant difference was found between these two MFCs. It appears that the BC MFC with a fully developed biofilm on the anodic surface, which would have been metabolising the incoming feedstock and converting it to by-products other than electricity (e.g. methane), had a similar effect on inactivation of core antigen of hepatitis B virus as CC MFC with the same position in the cascade. In contrast, the abiotic OC control without a biofilm had nearly no effect on HBcAg inactivation and almost 20 times higher and statistically significant LR values were observed for the corresponding CC MFC.

**Figure 4:**
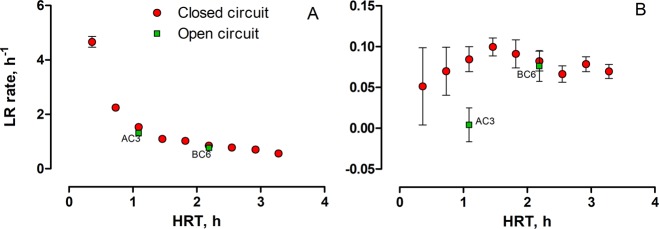
Dependence of log-reduction rate of: (**A**) surface (HBsAg) and (**B**) core (HBcAg) antigen concentrations on hydraulic retention time in open and closed circuit cascades. Data represent average ± SD.

To further investigate the kinetics and capability of virus inactivation in MFC reactors, the LR rate was calculated, as shown in Fig. 4. Surface antigen (Fig. 4A) displayed an exponential decay trend. Exceeding 1 hour of HRT significantly reduced the LR rate, reflecting the fact that the largest part of the surface antigen was inactivated within the first three CC MFCs.

The LR rate observed for core antigen (Fig. 4B) was significantly different to the HBsAg. The LR rate was increasing in each step of CC MFC cascade to reach a maximum value of 0.10 ± 0.01 h^−1^ after the 4^th^ (K4) MFC. Subsequently, a decreasing trend was observed, which stabilised in the last three MFCs. Therefore, the highest efficiency in virus (HBcAg) inactivation was observed for a retention time of approximately 1.5 hours. The above results indicate that such small scale MFCs would require at least 10 hours of HRT to reach a LR value of 1.0, suggesting a 90% core antigen inactivation.

**Figure 5:**
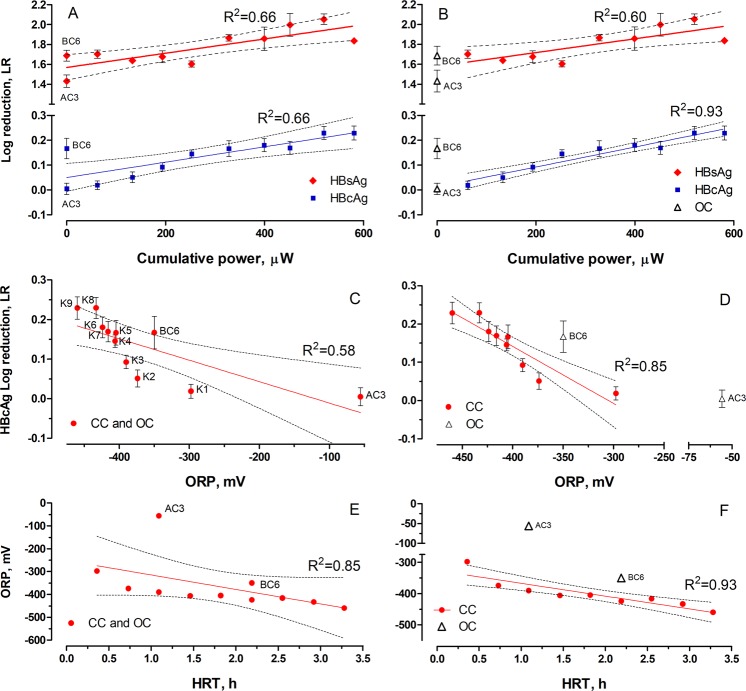
Linear regression models fitted to the experimental data. (**A**,**B**) Effect of cumulative power on both antigens when open circuit controls are included (**A**) and excluded (**B**) from the model. **C** and **D** – influence of ORP on log reduction of core antigen, when open circuit controls are included (**C**) and excluded (**D**). (**E**,**F**) Influence of HRT on ORP when the open circuit controls are included (**E**) and excluded (**F**). Dashed lines represent 95% confidence interval. Error bars represent standard deviation.

The regression analysis (Fig. 5) revealed a correlation between the ORP, HRT as well as cumulative power and LR. The cumulative power has been used since the inactivation efficiency was also dependent on the time of the exposure as demonstrated on Fig. 4. Regression analysis obtained for the dependence of LR on cumulative power displayed identical determination coefficients of R^2^ = 0.66 for both types of antigens (Fig. 5A). Excluding the AC and BC controls from the dataset resulted in great improvement of R2 value observed for core antigen (R^2^ = 0.93). Such results indicate that CC and OC MFCs do not belong to the same (statistical) populations due to the different reactions occurring in these units, although the current generation could have affected the physical-chemical parameters of the MFCs and therefore, inactivation of HBcAg. Furthermore detailed studies are needed to exploit the removal mechanisms.

Since the difference between OC and CC MFCs observed for surface antigen was not significant (Table 1), further regression models were only fitted to the HBcAg results. Excluding the AC and BC controls resulted in improvement of R^2^ value from 0.58 to 0.85 suggesting a good correlation of LR with redox potential of CC MFCs (Fig. 5A,B). Improving the R^2^ value by excluding the control samples also suggested that results observed for OC-MFCs could be considered as outliers as a different type of virus inactivation mechanism would have been taking place. This could partially be explained by the influence of HRT, and cumulative power on redox potential. In this case (Fig. 5E,F), excluding the OC MFCs resulted in an increase of the R^2^ from 0.85 to 0.93 and suggested that lower (more negative) ORPs reached for CC MFCs were directly related to the electroactive reactions. However, the ORP of biotic control (BC) was also very low (−350.0 mV) and was probably the main reason for such a high level of LR observed for BC6 MFC.

The low ORP and high LR observed for BC6 MFC could have resulted from the short-circuiting of electrodes through the earthenware membrane due to their high (14%) porosity^45^. Such a design of MFCs has previously resulted in deterioration of the cathodes^40^ and significant deposition of conductive salts on the surface of the outer cathode-electrodes^15^. It is assumed that the highly negative ORP along with the drop of the overall OCV values observed for BC-MFC OCV and CC-MFCs (Supplementary Fig. S1) indicate, that the BC biofilm was either somewhat “loaded”, carrying out electroactive reactions or fermentation. Previous work carried out with the same type of MFCs^15^ showed that OC MFCs matured in open circuit conditions had developed an electroactive community capable of producing up to 50% of power when compared to the most efficient closed circuit cells. Moreover, after 8–9 weeks of operation the capability of OC-MFCs in producing power exceeded the performance observed for CC-MFCs with deteriorated cathodes. Electroactive bacteria in OC MFCs were detected in other setups along with methanogens^46^. Considering the joint effect of high porosity of the separator and capability of OC biofilm to produce substantial current in polarisation experiments, it is possible to assume that the ceramic separator, saturated with highly conductive urine, acted as an electrochemical snorkel, allowing electrons to reach the cathode via alternative – to the circuit - routes. In such systems, the conductive bridging and mass transfer occurs even with no external wire applied between the electrodes^17,47,48^. Therefore, it is assumed that the BC-MFCs herein were inducing the LR due to the possible electroactive and methanogenic metabolisms of the biofilm developed within these MFCs and further studies to investigate the microbiome interactions with pathogens are certainly required.

The microbial consortia enriched with activated sludge are also rich in proteolytic enzymes which could have affected the virus inactivation in both types of biotic MFCs – BC and CC. Proteolytic enzymes are known for their efficient degradation of HBcAg^37^ and were found to be the main reason for the HBsAg degradation in faeces and sewage as described by Grabow *et al*.^42^.

It is noteworthy that abiotic control AC MFCs were significantly less efficient in inactivating core antigen. Therefore, HBcAg inactivation in CC and OC MFCs was solely the result of the metabolic activity of anaerobically respiring bacteria instead of abiotic reactions. The study of Nath *et al*. revealed that HBcAg is stable in the pH range of 5–10.5. Along with proteolytic activity, which was not monitored in this study, the HBcAg inactivation in MFCs therefore originated from the combined effect of HRT and redox potential induced by the electroactive metabolism of anaerobic microorganisms.

Furthermore, the lower than anticipated power output from these MFCs, predominantly because of the deteriorating cathode performance, is a factor that has to be considered for the weakened effect on the core antigen. These same MFCs have been previously reported to produce up to 150 μW, which is at least 2–3 times higher than the levels of power recorded in the current study. The hypothesis behind the killing or suppression of pathogens – bacteria^26,27^ as well as viruses – is that this is the result of antagonism from the incumbent biofilm, against alien-to-the-MFC biomolecules and lifeforms, for the available energy source in the environment. As already explained, pH and ORP play significant combined roles, but it is the unique ability of an electroactive community to metabolise the available carbon energy into electricity, via an electrode that would perhaps warrant successful competition against these incoming pathogenic perpetrators. Further studies will need to be carried out with the MFCs operating at, or close to, maximum power levels, for the given size/architecture, to be able to reach a conclusion as currently it is plausible to consider that the electrochemical snorkel effect^17,47,48^ (i.e. the possible ‘short-circuiting’ across the ceramic membrane) may have well played a key role in recording LR in surface and core antigen in the open circuit control MFCs.

This is the first report, in which the fate of two hepatitis B antigens was investigated in microbial fuel cell systems. The surface antigen (HBsAg) appeared to be much less stable and more susceptible to treatment in MFC cascades than core antigen (HBcAg). As a result, no significant difference between open circuit and closed circuit MFCs was observed. In contrast, the HBcAg was much more resistant to inactivation in the MFCs, leading to approximately 10 hours of hydraulic retention time required to reduce the core antigen quantity by 1 log-fold. A strong correlation was found between the cumulative power of the MFC cascade and log-reduction of core antigen, reflecting the influence of HRT, current generation and the resulting redox potential on inactivation of the HBcAg. Therefore, the present study postulates, that the risks related to the release of Hep B virus in sewage treatment systems, may be efficiently reduced in bioelectrochemical systems such as microbial fuel cells.

## Materials and Methods

### MFC design and operation

The MFCs were designed and manufactured in the same way as previously reported in detail^40^. In brief, single chamber air-cathode MFCs were constructed using cylinder ceramic earthenware membranes. The internal volume of empty MFCs was 11.4 mL. Anode electrodes were made from carbon veil −20 gC/m^2^ with a total surface area of 252 cm^2^. The cathodes were made of conductive graphite paint with a carbon loading of 35.02 mgC/cm^2^ reaching a total carbon loading for 24.18 cm^2^ cathode of 0.851 gC. A plain Ni-Cr wire (Ø0.45 mm) was used for connecting the electrodes with an external load. The 3D printed Nanocure® RCP30-resin and acrylic lids were used to cover the ceramic cylinder separators along with silicon gaskets. The 3D printed lid contained inlet and outlet tubes to facilitate continuous flow conditions.

### Experimental setup

The closed circuit (CC) MFC cascade consisted of 9 MFCs and the open circuit (OC) cascade of MFCs consisted of 6 MFCs (Fig. 1). Three of the MFCs operating in OC mode were designated as ‘abiotic control’ (AC) and the three downstream MFCs were designated as ‘biotic control’ (BC). Both CC and BC (open circuit) MFCs were inoculated with electroactive communities derived from anode anaerobic chambers of ongoing lab experiments. The abiotic control (AC) MFCs were not inoculated with bacteria and in addition were disinfected with 70% ethanol solution, followed by washing with sterile water and drying at 60 °C for 1 hour. The outlet of AC MFCs was the inlet of the BC MFCs. All of the MFCs in both cascades were separated by physical air gaps between the cells to avoid fluidic conductive connections between them.

Before testing the HBV antigen reduction in MFCs, the anodic biofilm had matured under steady state conditions and was operating for over 200 days under 1000 Ω (first 11 days) and 250 Ω (remaining period) external load. During the trial, all of the CC MFCs were connected to 250 Ω external load.

### Experimental procedure

During the trial, the MFCs were fed with neat human urine as the fuel, at a constant flow rate of 0.12 L/d by using a multichannel peristaltic pump (Watson Marlow, USA). Urine was supplemented with 50 ng/L active Hepatitis B Surface Antigen as well as active Hepatitis B Virus Core Antigen full length proteins (>95%, Abcam, UK). The feedstock supplemented with virus antigens was delivered to the MFC cascades for 18 hours, after which the samples were collected from the outlet of each sampling point, as shown in Fig. 1. Collected treated urine was immediately analysed using a 96-well microplate QuickTiter™ Hepatitis B Surface Antigen (HBsAg) ELISA Kit and QuickTiter™ Hepatitis B Core Antigen (HBVcAg) ELISA Kit according to the manufacturer specifications (Cell Biolabs Inc. CA, US). Each microplate was analysed using a FLUOstar OPTIMA microplate reader at 450 nm wavelength (BMG Labtech, Germany). The pH and ORP were measured with Orion Dual Star pH meter (Thermo Fisher Scientific, USA).

The log reduction (LR) was determined by using the following formula:

$$LR=\,\mathrm{log}(\frac{A}{B})$$

where:

A – concentration of the antigen before treatment in MFCs (inlet to the cascade),

B – concentration of the antigen after treatment in the MFCs (MFC outlet(s)).

The standard deviation was calculated as described by Zelver *et al*.^49^:

$$S{D}_{LR}=[({S}_{A}^{2}/{n}_{A})+({S}_{B}^{2}/{n}_{B})]$$

where:

S_A_ and S_B_ - the sample standard deviations of the log reduction values for samples before and after treatment, respectively;

n_A_ and n_B_ – number of replicates in population before and after treatment, respectively.

### Data logging and processing

The real time temporal potential of all MFCs in cascades was monitored using Picolog ADC-24 Data Logger (Pico Technologies, UK). The sampling rate of data logging was set up to 3 minutes. The current was calculated according to Ohm’s law: I = V/R, where V is the measured voltage in Volts (V) and R is the value of the external resistance. The power output P in Watts (W) was calculated using equation: P = I × V. Experimental data were processed using Microsoft Excel 2010, analysed using RGui statistical environment (Shapiro-Wilk test, t-test, Wilcoxon-Matt-Whitney test) and plotted by GraphPad Prism 5.0 software.

## Supplementary information

Supplementary information

## Data Availability

All data generated or analysed during this study are included in this published article (and its Supplementary Information files).
