# Supplementary material for: Removal of Hepatitis B virus surface HBsAg and core HBcAg antigens using microbial fuel cells producing electricity from human urine
Source: Sci Rep. 2019 Aug 13;9:11787. doi: 10.1038/s41598-019-48128-x (PMC6692344; doi:10.1038/s41598-019-48128-x)
Supplement: Supplementary file 1 — Supplementary information [file 41598_2019_48128_MOESM1_ESM.docx]

**Removal of Hepatitis B virus surface HBsAg and core HBcAg antigens using microbial fuel cells producing electricity from human urine**

Grzegorz Pasternak^a,b^, John Greenman^a^, Ioannis Ieropoulos^*a^

^a^Bristol BioEnergy Centre, Bristol Robotics Laboratory, University of the West of England, Coldharbour Lane, BS16 1QY Bristol, UK.

^b^Faculty of Chemistry, Wroclaw University of Science and Technology, Wyb. Wyspiańskiego 27, 50-370 Wrocław, Poland.

^*^Corresponding author: [ioannis.ieropoulos@brl.ac.uk](mailto:ioannis.ieropoulos@brl.ac.uk), +44 (0)117 32 86318.

**Supporting information**


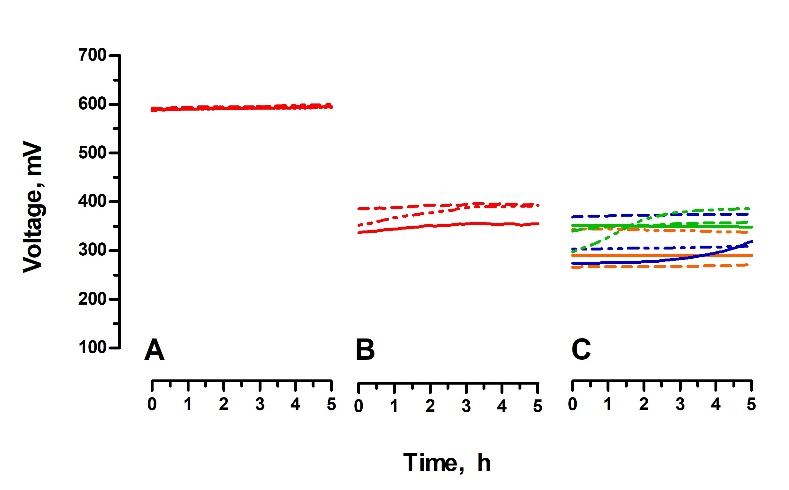


**Supplementary Figure S1.** Comparison of open circuit voltage (OCV) of: A - BC-MFCs (BC4-BC6) shortly after inoculation; B - during the killing rate trials and after observed deterioration of cathodes; C - OCV observed for CC-MFCs shortly before the virus inactivation rate trial.
